# 2,2′-(Ethane-1,2-di­yl)bis­(1*H*-benzimidazole)

**DOI:** 10.1107/S1600536812013839

**Published:** 2012-04-06

**Authors:** Xu Chen, Xiao-Yong Wu, Guo-Liang Zhao

**Affiliations:** a Zhejiang Normal University Xingzhi College, Jinhua, Zhejiang 321004, People’s Republic of China; b College of Chemistry and Life Sciences, Zhejiang Normal University, Jinhua 321004, Zhejiang, People’s Republic of China

## Abstract

The complete mol­ecule of the title compound, C_16_H_14_N_4_, is generated by crystallographic inversion symmetry. In the crystal, mol­ecules are linked by N—H⋯N hydrogen bonds, generating (001) sheets. Weak aromatic π–π stacking inter­actions [centroid–centroid distances = 3.7383 (13) and 3.7935 (14) Å] are also observed.

## Related literature

For background to metal-organic frameworks, see: van Albada *et al.* (2007 ▶). For the synthesis, see: Wang & Joulli (1957 ▶).
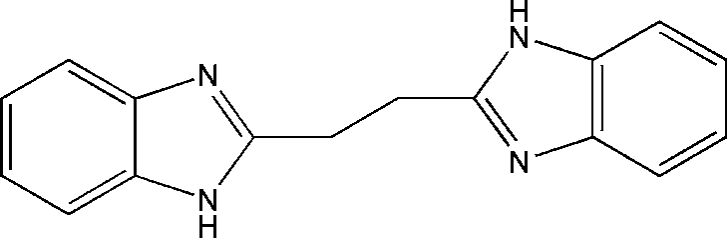


## Experimental

### 

#### Crystal data

C_16_H_14_N_4_
*M* *_r_* = 262.31
Orthorhombic, 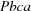

*a* = 8.4295 (18) Å
*b* = 9.924 (2) Å
*c* = 15.351 (4) Å
*V* = 1284.2 (5) Å^3^
*Z* = 4
Mo *K*α radiation
μ = 0.08 mm^−1^
*T* = 296 K
0.32 × 0.25 × 0.19 mm

#### Data collection

Bruker APEXII CCD diffractometer
Absorption correction: multi-scan (*SADABS*; Sheldrick, 1996 ▶) *T*_min_ = 0.975, *T*_max_ = 0.984
10702 measured reflections
1475 independent reflections
966 reflections with *I* > 2σ(*I*)
*R*_int_ = 0.052

#### Refinement

*R*[*F* ^2^ > 2σ(*F* ^2^)] = 0.045
*wR*(*F* ^2^) = 0.122
*S* = 1.02
1475 reflections
91 parameters
H-atom parameters constrained
Δρ_max_ = 0.15 e Å^−3^
Δρ_min_ = −0.21 e Å^−3^

### 

Data collection: *APEX2* (Bruker, 2006 ▶); cell refinement: *SAINT* (Bruker, 2006 ▶); data reduction: *SAINT*; program(s) used to solve structure: *SHELXS97* (Sheldrick, 2008 ▶); program(s) used to refine structure: *SHELXL97* (Sheldrick, 2008 ▶); molecular graphics: *SHELXTL* (Sheldrick, 2008 ▶); software used to prepare material for publication: *SHELXL97*.

## Supplementary Material

Crystal structure: contains datablock(s) I, global. DOI: 10.1107/S1600536812013839/hb6688sup1.cif

Structure factors: contains datablock(s) I. DOI: 10.1107/S1600536812013839/hb6688Isup2.hkl

Supplementary material file. DOI: 10.1107/S1600536812013839/hb6688Isup3.cml

Additional supplementary materials:  crystallographic information; 3D view; checkCIF report

## Figures and Tables

**Table 1:** Hydrogen-bond geometry (Å, °)

*D*—H⋯*A*	*D*—H	H⋯*A*	*D*⋯*A*	*D*—H⋯*A*
N2—H2*B*⋯N1^i^	0.86	2.04	2.8568 (18)	159

Symmetry code: (i) 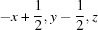.

**Fig. 1.**
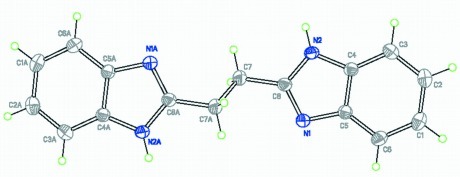
The molecular structure of the title compound with displacement ellipsoids drawn at the 30% probability level. Atoms with suffix A are at the symmetry position (1–x, 1–y, –z).

**Fig. 2.**
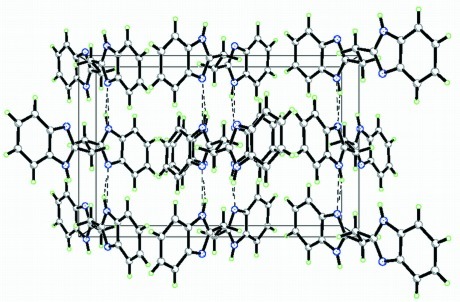
The stacking plot of the title compound, showing H-bond interactions (dashed lines) and π–π stacking interactions.

## supplementary crystallographic information

### Comment

We present a polyaromatic compoud (1) that contains multiple functional groups that can develop a series of metal-organic frameworks with potential applications (e.g. van Albada *et al.* (2007)). The molecular struture of (1), shown in Fig.1, consists of two symmetrical benzimidazole rings. The two benzimidazolyl rings are nearly parallel, witha dihedral angle of 2.645 (6)° between them. Molecules are linked *via* a network of hydrogen bonds (N2—H2B···N1; Table 2). π–π stacking interactions are observed between nearly parallel benzimidazolyl benzene rings. The centroid-to-centroid distance between C1—C6 benzene rings is 3.7379 Å, while between C1A—C6A benzene rings it is 3.7944 Å (the symmetry operation: -*x* + 1,-*y*,-*z*). The hydrogen bonds and π–π weak non-covalent interactions lend stability to the structure. The stacking plot of this compound was shown in Fig. 2.

### Experimental

ο-Phenylenediamine (1.081 g, 10 mmol) and succinic acid (0.590 g, 5 mmol) were refluxed for 4 h in 30 ml of 10% hydrochloric acid solution. The reaction mixture was cooled to room temperature, the precipitation that formed was filtered and then recrystallized from water. The colourless crystals were obtained from water after a week (Wang *et al.*, 1957).

### Refinement

The H atoms bonded to C and N atoms were positioned geometrically and refined using a riding model [aliphatic C—H =0.96 Å (*U*_iso_(H) = 1.5*U*_eq_(C)), aromatic C—H = 0.93 Å (*U*_iso_(H) = 1.2*U*_eq_(C)) and N—H = 0.86 Å with *U*_iso_(H) = 1.2*U*_eq_(N).

### Crystal data

**Table:**

C_16_H_14_N_4_	*F*(000) = 552
*M**_r_* = 262.31	*D*_x_ = 1.357 Mg m^−^^3^
Orthorhombic, *P**b**c**a*	Mo *K*α radiation, λ = 0.71073 Å
Hall symbol: -P 2ac 2ab	θ = 2.7–27.6°
*a* = 8.4295 (18) Å	µ = 0.08 mm^−^^1^
*b* = 9.924 (2) Å	*T* = 296 K
*c* = 15.351 (4) Å	Block, colourless
*V* = 1284.2 (5) Å^3^	0.32 × 0.25 × 0.19 mm
*Z* = 4	

### Data collection

**Table:**

Bruker APEXII CCD diffractometer	1475 independent reflections
Radiation source: fine-focus sealed tube	966 reflections with *I* > 2σ(*I*)
Graphite monochromator	*R*_int_ = 0.052
phi and ω scans	θ_max_ = 27.6°, θ_min_ = 2.7°
Absorption correction: multi-scan (*SADABS*; Sheldrick, 1996)	*h* = −10→10
*T*_min_ = 0.975, *T*_max_ = 0.984	*k* = −12→12
10702 measured reflections	*l* = −19→16

### Refinement

**Table:**

Refinement on *F*^2^	Primary atom site location: structure-invariant direct methods
Least-squares matrix: full	Secondary atom site location: difference Fourier map
*R*[*F*^2^ > 2σ(*F*^2^)] = 0.045	Hydrogen site location: inferred from neighbouring sites
*wR*(*F*^2^) = 0.122	H-atom parameters constrained
*S* = 1.02	*w* = 1/[σ^2^(*F*_o_^2^) + (0.0554*P*)^2^ + 0.2274*P*] where *P* = (*F*_o_^2^ + 2*F*_c_^2^)/3
1475 reflections	(Δ/σ)_max_ < 0.001
91 parameters	Δρ_max_ = 0.15 e Å^−^^3^
0 restraints	Δρ_min_ = −0.21 e Å^−^^3^

### Special details

**Table:**

Geometry. All e.s.d.'s (except the e.s.d. in the dihedral angle between two l.s. planes) are estimated using the full covariance matrix. The cell e.s.d.'s are taken into account individually in the estimation of e.s.d.'s in distances, angles and torsion angles; correlations between e.s.d.'s in cell parameters are only used when they are defined by crystal symmetry. An approximate (isotropic) treatment of cell e.s.d.'s is used for estimating e.s.d.'s involving l.s. planes.
Refinement. Refinement of *F*^2^ against ALL reflections. The weighted *R*-factor *wR* and goodness of fit *S* are based on *F*^2^, conventional *R*-factors *R* are based on *F*, with *F* set to zero for negative *F*^2^. The threshold expression of *F*^2^ > σ(*F*^2^) is used only for calculating *R*-factors(gt) *etc*. and is not relevant to the choice of reflections for refinement. *R*-factors based on *F*^2^ are statistically about twice as large as those based on *F*, and *R*- factors based on ALL data will be even larger.

### Fractional atomic coordinates and isotropic or equivalent isotropic displacement parameters (Å^2^)

**Table:**

	*x*	*y*	*z*	*U*_iso_*/*U*_eq_	
N2	0.18376 (15)	0.39276 (12)	0.06131 (8)	0.0378 (4)	
H2B	0.1935	0.3084	0.0498	0.045*	
N1	0.23667 (16)	0.61293 (12)	0.06218 (9)	0.0402 (4)	
C5	0.09915 (18)	0.58969 (15)	0.11024 (10)	0.0357 (4)	
C8	0.28216 (19)	0.49246 (14)	0.03497 (11)	0.0375 (4)	
C4	0.06495 (18)	0.45137 (15)	0.11012 (10)	0.0354 (4)	
C7	0.42738 (19)	0.46475 (17)	−0.01726 (12)	0.0452 (4)	
H7A	0.4092	0.4932	−0.0769	0.054*	
H7B	0.4467	0.3684	−0.0178	0.054*	
C3	−0.0631 (2)	0.39827 (18)	0.15503 (11)	0.0475 (5)	
H3A	−0.0860	0.3066	0.1537	0.057*	
C6	0.0040 (2)	0.67805 (16)	0.15747 (11)	0.0461 (5)	
H6A	0.0247	0.7701	0.1581	0.055*	
C2	−0.1552 (2)	0.48740 (18)	0.20187 (12)	0.0524 (5)	
H2A	−0.2416	0.4551	0.2333	0.063*	
C1	−0.1215 (2)	0.62512 (18)	0.20317 (12)	0.0520 (5)	
H1A	−0.1856	0.6824	0.2357	0.062*	

### Atomic displacement parameters (Å^2^)

**Table:**

	*U*^11^	*U*^22^	*U*^33^	*U*^12^	*U*^13^	*U*^23^
N2	0.0346 (8)	0.0281 (6)	0.0506 (8)	0.0003 (5)	0.0010 (6)	−0.0046 (6)
N1	0.0373 (8)	0.0317 (7)	0.0515 (8)	−0.0013 (5)	0.0053 (6)	−0.0039 (6)
C5	0.0323 (9)	0.0347 (8)	0.0402 (9)	0.0015 (7)	−0.0014 (7)	0.0005 (6)
C8	0.0328 (9)	0.0329 (8)	0.0469 (9)	−0.0014 (6)	−0.0024 (7)	−0.0044 (7)
C4	0.0326 (9)	0.0330 (8)	0.0407 (9)	0.0013 (6)	−0.0027 (7)	−0.0005 (6)
C7	0.0343 (9)	0.0450 (9)	0.0563 (11)	−0.0019 (7)	0.0050 (8)	−0.0102 (8)
C3	0.0458 (11)	0.0432 (9)	0.0537 (10)	−0.0071 (8)	0.0034 (9)	0.0023 (8)
C6	0.0473 (11)	0.0371 (9)	0.0541 (11)	0.0069 (8)	0.0020 (9)	−0.0036 (7)
C2	0.0435 (11)	0.0626 (12)	0.0512 (11)	−0.0013 (9)	0.0105 (9)	0.0027 (9)
C1	0.0458 (11)	0.0571 (11)	0.0529 (11)	0.0130 (9)	0.0083 (9)	−0.0057 (9)

### Geometric parameters (Å, º)

**Table:**

N2—C8	1.3530 (19)	C7—H7A	0.9700
N2—C4	1.3795 (19)	C7—H7B	0.9700
N2—H2B	0.8600	C3—C2	1.379 (2)
N1—C8	1.3233 (18)	C3—H3A	0.9300
N1—C5	1.393 (2)	C6—C1	1.374 (2)
C5—C6	1.392 (2)	C6—H6A	0.9300
C5—C4	1.403 (2)	C2—C1	1.396 (2)
C8—C7	1.489 (2)	C2—H2A	0.9300
C4—C3	1.385 (2)	C1—H1A	0.9300
C7—C7^i^	1.506 (3)		
			
C8—N2—C4	107.40 (12)	C8—C7—H7B	109.0
C8—N2—H2B	126.3	C7^i^—C7—H7B	109.0
C4—N2—H2B	126.3	H7A—C7—H7B	107.8
C8—N1—C5	104.99 (12)	C2—C3—C4	117.01 (16)
C6—C5—N1	130.61 (14)	C2—C3—H3A	121.5
C6—C5—C4	119.91 (15)	C4—C3—H3A	121.5
N1—C5—C4	109.41 (13)	C1—C6—C5	117.96 (15)
N1—C8—N2	112.88 (14)	C1—C6—H6A	121.0
N1—C8—C7	125.11 (14)	C5—C6—H6A	121.0
N2—C8—C7	122.00 (13)	C3—C2—C1	121.40 (17)
N2—C4—C3	132.51 (15)	C3—C2—H2A	119.3
N2—C4—C5	105.32 (13)	C1—C2—H2A	119.3
C3—C4—C5	122.14 (15)	C6—C1—C2	121.57 (16)
C8—C7—C7^i^	113.14 (16)	C6—C1—H1A	119.2
C8—C7—H7A	109.0	C2—C1—H1A	119.2
C7^i^—C7—H7A	109.0		
			
C8—N1—C5—C6	176.72 (17)	N1—C5—C4—C3	178.13 (14)
C8—N1—C5—C4	−0.20 (18)	N1—C8—C7—C7^i^	46.1 (3)
C5—N1—C8—N2	0.41 (18)	N2—C8—C7—C7^i^	−132.3 (2)
C5—N1—C8—C7	−178.13 (15)	N2—C4—C3—C2	176.40 (16)
C4—N2—C8—N1	−0.47 (19)	C5—C4—C3—C2	−1.2 (2)
C4—N2—C8—C7	178.12 (14)	N1—C5—C6—C1	−176.40 (16)
C8—N2—C4—C3	−177.62 (17)	C4—C5—C6—C1	0.2 (2)
C8—N2—C4—C5	0.31 (17)	C4—C3—C2—C1	0.6 (3)
C6—C5—C4—N2	−177.37 (14)	C5—C6—C1—C2	−0.9 (3)
N1—C5—C4—N2	−0.07 (17)	C3—C2—C1—C6	0.4 (3)
C6—C5—C4—C3	0.8 (2)		

Symmetry code: (i) −*x*+1, −*y*+1, −*z*.

### Hydrogen-bond geometry (Å, º)

**Table:**

*D*—H···*A*	*D*—H	H···*A*	*D*···*A*	*D*—H···*A*
N2—H2*B*···N1^ii^	0.86	2.04	2.8568 (18)	159

Symmetry code: (ii) −*x*+1/2, *y*−1/2, *z*.
